# The Lnk/SH2B adaptor provides a fail-safe mechanism to establish the Insulin receptor-Chico interaction

**DOI:** 10.1186/1478-811X-11-26

**Published:** 2013-04-16

**Authors:** Isabel Almudi, Ingrid Poernbacher, Ernst Hafen, Hugo Stocker

**Affiliations:** 1 Institute of Molecular Systems Biology, ETH Zürich, Wolfgang-Pauli-Strasse 16, Zürich 8093, Switzerland; 2 Current address: Department of Health and Life Sciences, Oxford Brookes University, Gipsy lane, Oxford OX3 0BP, UK; 3 Current address: MRC National Institute for Medical Research, The Ridgeway, Mill Hill, London NW7 1AA, UK

## Abstract

**Background:**

Insulin/insulin-like growth factor signalling (IIS) has been described as one of the major pathways involved in growth control and homeostasis in multicellular organisms. Whereas its core components are well established, less is known about the molecular functions of IIS regulators. The adaptor molecule Lnk/SH2B has been implicated in IIS but the mechanism by which it promotes IIS activity has remained enigmatic.

**Results:**

In this study, we analyse genetic and physical interactions among InR, Chico and Lnk in *Drosophila* tissues. FRET analysis reveals in vivo binding between all three molecules. Genetically, Lnk acts upstream of Chico. We demonstrate that Chico’s plasma membrane localisation is ensured by both its PH domain and by the interaction with Lnk. Furthermore, Lnk is able to recruit an intracellular InR fragment to the membrane.

**Conclusions:**

Thus, by acting as a scaffolding molecule that ensures InR and Chico enrichment at the membrane, Lnk provides a fail-safe mechanism for IIS activation.

## Background

The Insulin/insulin-like growth factor signalling (IIS) pathway has emerged in the last decade as one of the major signalling pathways involved in the control of growth, body size and homeostasis in multicellular organisms [1-4].

The main intracellular components of IIS in *Drosophila* are Chico, the homologue of the Insulin Receptor Substrates (IRS), the lipid kinase phosphoinositide 3-kinase (PI3K), the lipid phosphatase PTEN and the serine-threonine kinase dAkt/PKB [5-10]. These intracellular signalling components need to be recruited to the cortical membrane to regulate signalling activity [5,7,11-13]. In addition to the core components, regulators such as Susi [14], Steppke [15] and Lnk [16,17] modulate IIS activity.

The Lnk adaptor protein has been identified in an unbiased screen as a component of the pathway based on the reduced body size and lipid accumulation observed in *lnk* mutant flies [17]. Mutations in the *lnk* locus were able to rescue the overgrowth phenotype caused by overexpression of InR, but not to suppress the overgrowth promoted by high activity of PI3K, suggesting that Lnk acts between InR and PI3K in the IIS pathway [17]. Moreover, phosphorylation of PKB and tGPH reporter localisation [18], both readouts of IIS pathway activity, were impaired in *lnk* mutants [17]. Lnk is the unique *Drosophila* member of the SH2B protein family. This protein family is characterised by several conserved domains: the N-terminal proline-rich stretch, a pleckstrin homology (PH) domain, a Src homology 2 (SH2) domain, and a C-terminal c-Cbl recognition motif [19-21]. Alleles with inactive PH or SH2 domains have similar phenotypes to those carrying premature stop codons, suggesting that both domains are essential for Lnk activity [17].

Here we study the molecular function of Lnk in *Drosophila*. We first apply the Förster Resonance Energy Transfer (FRET) technique in *Drosophila* larvae to demonstrate that Lnk binds to Chico and InR in vivo. Second, we show that Lnk functions upstream of Chico. Finally, we demonstrate that Lnk ensures proper localisation of InR and Chico to trigger IIS.

## Results and discussion

### InR, Chico and Lnk physically interact in vivo

Previous studies have demonstrated that a mammalian homologue of Lnk, SH2B, co-immunoprecipitates with the mammalian InR in cultured cells [20,22]. Moreover, Lnk and Chico have been shown to co-immunoprecipitate in *Drosophila* S2 cells [16]. However, the interactions between the three molecules in vivo have remained elusive. Therefore, we set out to investigate the binding between InR, Chico and Lnk using FRET in *Drosophila* tissues. We generated constructs to drive expression of tagged InR, Chico and Lnk proteins based on the UAS/Gal4 system [23]. In order to analyse the physical interactions between the three molecules in vivo, we modified phiC31 UASattB vectors to C-terminally tag the expressed proteins with Cyan Fluorescent Protein (CFP) and monomeric Red Fluorescent Protein (RFP), respectively (see Methods).

**Figure 1:**
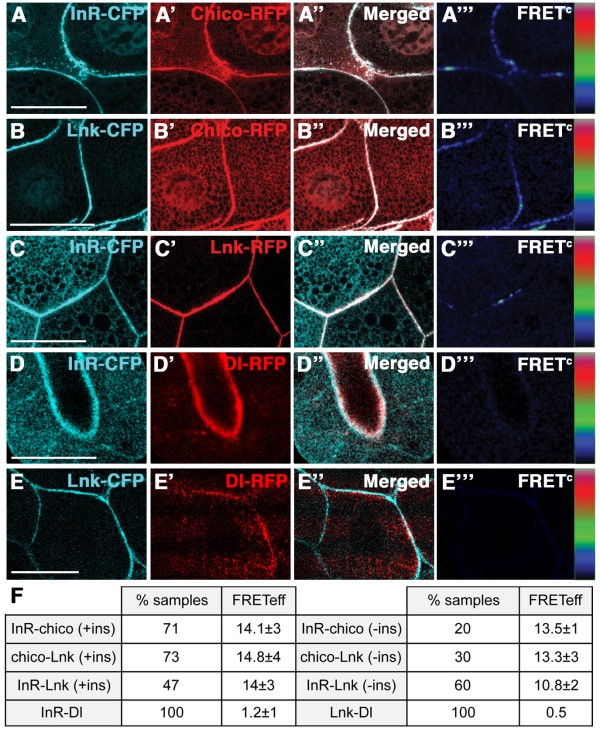
**In vivo FRET analysis reveals physical interactions among Lnk, Chico and InR.** (**A**-A”’) Chico-RFP (A’) and InR-CFP (**A**) strongly co-localise in salivary glands (A”). FRET^c^ shows regions with high energy transfer between CFP and RFP (colour code: black - low intensities, red - high intensities). (**B**-B”’) Lnk-CFP (**B**) and Chico-RFP (B’) co-localise (B”) showing positive FRET^c^ (B”’). (**C**-C”’) InR-CFP (**C**) and Lnk-RFP (C’) localisation (C”) and FRET (C”’) at the cortical membrane. (**D**-D”’) InR-CFP (**D**) and Dl-RFP (D’) do not exhibit energy transfer (D”’). (**E**-E”’) Lnk-CFP (**E**) and Dl-RFP (E’) do not show positive FRET^c^ (E”’). (**F**) Table showing data from FRET analyses. Number of samples analysed: (**A**) n = 21 (+ins) and n = 10 (−ins), (**B**) n = 20 (+ins) and n = 10 (−ins), (**C**) n = 17 (+ins) and n = 11 (−ins), (**D**) n = 15, (**E**) n = 14. Scale bars represent 50 μm.

We first assessed the FRET efficiency (FRETeff) between the known binding partners InR and Chico by overexpressing *UAS-InR-CFP* and *UAS-chico-RFP* with *hsp-Gal4* in larval salivary glands. We chose the salivary glands because of the large cell size. FRET between CFP and RFP (FRETeff = 14.1 ± 3%) was observed in 71% of the tissue samples examined after insulin stimulation (Figure 1A and F). Interestingly, we detected FRET between the two molecules only in 20% of the samples in the absence of insulin (FRETeff = 13.5 ± 1%), indicating that InR-Chico binding is stimulated by insulin as previously reported [24,25].

We next investigated the binding of Lnk to Chico. In *lnk-CFP/chico-RFP* salivary glands, FRETeff was 14.8 ± 4% in 73% of the samples upon insulin stimulation (Figure 1B and F). By contrast, non-stimulated tissue samples showed a reduction in Lnk-Chico interaction (FRETeff = 13.3 ± 3% in 30% of samples), suggesting that, like Chico-InR binding, the Chico-Lnk interaction depends on insulin stimulation.

We also tested whether Lnk can directly bind to InR and found positive energy transfer in 53% of the salivary glands examined (FRETeff = 14 ± 3%; Figure 1C and F). Remarkably, when we analysed FRET between Lnk and InR in the absence of insulin, FRETeff = 10.8 ± 2% was observed in 60% of the samples, implying that the interaction between Lnk and InR occurs, at least partially, in an insulin-independent way. We did not detect energy transfer between Delta-RFP (Dl-RFP) [26,27] and InR-CFP (Figure 1D and F, FRETeff < 3%) or Lnk-CFP (Figure 1E and F, FRETeff < 3%), excluding that InR-CFP and Lnk-CFP unspecifically interact with membrane-bound proteins. Thus, Lnk acts as a direct binding partner of both InR and Chico in *Drosophila* tissues. Interestingly, the Chico-Lnk and Chico-InR interactions are insulin dependent, whereas Lnk and InR are capable of interacting without insulin stimulation. These differences in response to insulin may reflect specific functions of the molecules under examination.

### Lnk facilitates the recruitment of Chico to the membrane

**Figure 2:**
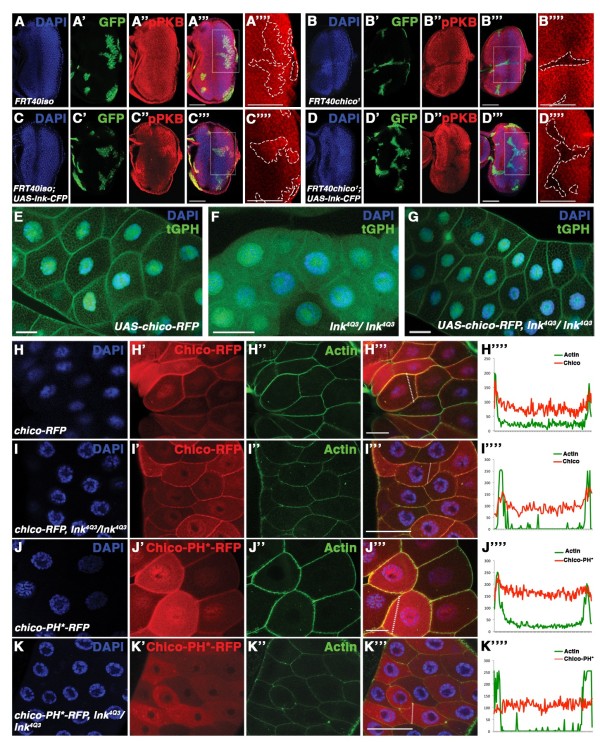
**Lnk acts upstream of Chico ensuring its proper localisation.** (**A-D**) Low phospho-PKB levels in *chico*^*−/−*^ MARCM clones are not rescued by overexpression of *lnk-CFP*. Clones are marked by GFP expression (’ panels) and outlined by a dashed line in the detail pictures (”” panels). Phospho-PKB staining is shown in ” panels. (**A**-A””) control clones. (**B**-B””) Levels of phospho-PKB (B”) are decreased in *chico*^*−/−*^ clones. (**C**-C””) Overexpression of *lnk-CFP* within control clones promotes high levels of phospho-PKB (C”). (**D**-D””) *lnk-CFP* overexpression within *chico*^*−/−*^ clones does not rescue low levels of phospho-PKB (D”). (**E-G**) *Chico* overexpression restores localisation of the tGPH reporter at the membrane in *lnk*^*−/−*^ salivary glands. (**E**) tGPH reporter localises at the membrane in wild-type salivary glands. (**F**) *lnk*^*4Q3*^ salivary glands show diffuse tGPH reporter signal in the cytoplasm. (**G**) tGPH reporter is recovered to the membrane of *lnk*^*4Q3*^ tissue by overexpressing *chico.* (**H**-**K**) Subcellular localisation of RFP-tagged Chico. The RFP signal is shown in ’ panels. Actin staining marks the cellular cortex in ” panels. Merged pictures are shown in ”’ panels. Signal intensities along the lines indicated in the ”’ panels are displayed in ”” panels. Quantifications in Additional file 1: Figure S1A. (**H**-H””) Cortical localisation of Chico-RFP in a wild-type situation. (**I**-I””) Chico-RFP in a *lnk* mutant background. (**J**-J””) A PH domain mutant form of Chico localises at the cortical membrane. (**K**-K””) Chico-PH*-RFP in a *lnk* mutant background. Number of samples analysed: (**A**) n=9, (**B**) n=9, (**C**) n = 10, (**D**) n = 12, (**E**) n=5, (**F**) n=5, (**G**) n=6, (**H**) n=6, (**I**) n=9, (**J**) n=6, (**K**) n = 6. Scale bars represent 50 μm.

Once we established that Lnk is forming a complex with InR and Chico, we wondered about the molecular function of Lnk. To address whether Lnk is acting in parallel to Chico, we first investigated whether overexpression of *lnk* was able to rescue the decrease of IIS activity in *chico* mutant cells. The MARCM (mosaic analysis with a repressible cell marker) system was used to generate clonal populations of *chico* mutant cells that overexpress *lnk-CFP* and are marked by the expression of GFP. We induced MARCM clones in eye imaginal discs and performed immunostainings against phospho-PKB to assess IIS activity. Whereas overexpression of *lnk-CFP* was able to increase phospho-PKB levels in clones (marked by GFP) in comparison to wild-type tissue (Figure 2A and C), we did not observe a rescue of the low phospho-PKB levels in *chico*^*−/−*^ clones upon *lnk-CFP* overexpression (Figure 2B and D).

For the inverse experiment, we used *lnk* mutant salivary glands and analysed IIS pathway activity by means of a tGPH reporter [18]. In *lnk* mutant salivary glands, we observed the tGPH reporter mainly in the cytoplasm, which indicates low IIS activity (Figure 2F, [17]). By contrast, overexpression of *chico-RFP* in *lnk* mutant salivary glands resulted in localisation of the tGPH reporter to the plasma membrane, reflecting high IIS activity (Figure 2E and G). Thus, overexpression of *chico-RFP* counteracts the loss of *lnk* function, suggesting that Chico acts downstream of Lnk.

To analyse whether Lnk facilitates the localisation of Chico, we first studied the localisation of Chico-RFP in *lnk* mutant salivary glands. We determined the intensity of the Chico-RFP signal at the membrane and in the cytoplasm to assess the relative amounts of protein in these compartments under different experimental conditions (see Methods). The membrane localisation of Chico-RFP was only slightly reduced in *lnk* mutant tissue in comparison to wild-type tissue (Figure 2H, I and Additional file 1: Figure S1A), probably due to the PH domain of Chico. In fact, when we expressed a PH-domain mutated form of Chico (Chico-PH*-RFP) in a *lnk* mutant background, we noticed a relocalisation of Chico-PH*-RFP from the membrane to the cytoplasm (Figure 2K and Additional file 1: Figure S1A). By contrast, Chico-PH*-RFP showed significant localisation to the plasma membrane in wild-type tissue (Figure 2J and Additional file 1: Figure S1A), indicating that Lnk is sufficient to substitute the function of the PH domain in Chico-PH*-RFP. Thus, Lnk provides a redundant means to properly localise Chico at the cortical membrane.

### Lnk ensures InR enrichment at the cortical membrane

Our genetic and localisation data of Chico and Lnk might seem contradictory to previous genetic interaction experiments between *chico* and *lnk* mutants; if Lnk was only required for proper Chico function, *chico; lnk* double mutants should display similar phenotypes to *chico* single mutants. However, whereas the single mutants are reduced in size but viable, the *chico; lnk* double mutants turned out to be lethal [17]. One way to reconcile these findings is to propose an additional direct function of Lnk on InR.

**Figure 3:**
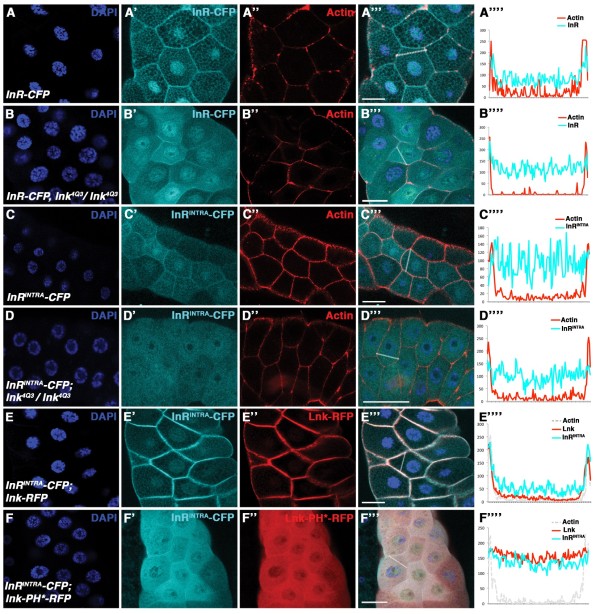
**Lnk recruits the intracellular part of InR to the membrane.** CFP-tagged InR (fragments) are shown in ’ panels. Actin staining marks the cellular cortex in ” panels except for E and F where Lnk-RFP is shown in the ” panels. Merged pictures are shown in ”’ panels. Signal intensities measured along the indicated lines in ”’ panels are displayed in ”” panels. Quantifications in Additional file 1: Figure S1B. (**A-B**) InR-CFP is substantially reduced at the cellular cortex in *lnk* mutant tissue (**B**-B””) in comparison to wild-type salivary glands (**A**-A””). (**C**-C””) Localisation of InR^INTRA^-CFP (C’) in wild-type tissue. (**D**-D””) InR^INTRA^-CFP in *lnk*^*−/−*^ tissue. (**E**-E””) Co-localisation of InR^INTRA^-CFP (E’) and Lnk-RFP (E”) at the cellular membrane. (**F**-F””) Co-localisation of InR^INTRA^-CFP (F’) and Lnk-PH*-RFP (F”) in the cytoplasm. Number of samples analysed: (**A**) n=5, (**B**) n=6, (**C**) n=6, (**D**) n=7, (**E**) n=6, (**F**) n = 5. Scale bars represent 50 μm.

We analysed InR-CFP localisation in *lnk* mutant salivary glands to test whether Lnk facilitates InR localisation. In contrast to InR-CFP in wild-type tissue, where InR-CFP was located mainly at the cortical membrane (Figure 3A and Additional file 1: Figure S1B), InR-CFP was decreased at the membrane in a *lnk* mutant background (Figure 3B and Additional file 1: Figure S1B). However, a fraction of InR-CFP still localised at the plasma membrane, most likely due to InR’s transmembrane domain. We next generated an intracellular InR construct (hereafter InR^INTRA^) containing the intracellular domain of InR (beginning with the kinase domain) fused to CFP at the C-terminus (InR^INTRA^-CFP). InR^INTRA^-CFP membrane localisation was reduced already in a wild-type background (Figure 3C and Additional file 1: Figure S1B), whereas full-length InR-CFP showed a similar effect only in *lnk* mutant salivary glands. In a *lnk* mutant background, cortical accumulation of InR^INTRA^-CFP was reduced more strongly (Figure 3D and Additional file 1: Figure S1B), further supporting the role of Lnk in locking InR to the membrane. Moreover, overexpression of *InR*^*INTRA*^*-CFP* together with *lnk-RFP* restored cortical localisation of InR^INTRA^-CFP, showing both molecules at the plasma membrane (Figure 3E and Additional file 1: Figure S1B). The membrane localisation was essentially abolished when a PH domain mutant version of Lnk was expressed (Figure 3F and Additional file 1: Figure S1B). These experiments strongly suggest that Lnk contributes to the cortical localisation of InR by interacting with the intracellular part of InR.

**Figure 4:**
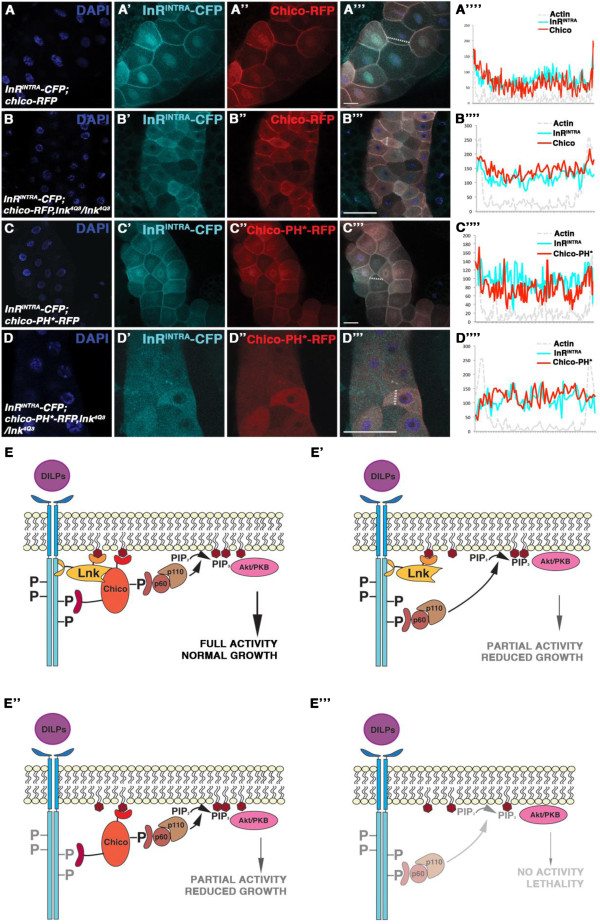
**Lnk reinforces membrane localisation of Chico-RFP and InR**^**INTRA**^**-CFP.** CFP-tagged InR^INTRA^ is shown in ’ panels, RFP-tagged Chico versions in ” panels. Merged pictures are shown in ”’ panels. Signal intensities measured along the indicated lines in ”’ panels are displayed in ”” panels. Quantifications in Additional file 1: Figure S1C. (**A**-A””) InR^INTRA^-CFP (A’) and Chico-RFP (A”) in a wild-type background. (**B**-B””) InR^INTRA^-CFP (B’) and Chico-RFP (B”) in *lnk*^*4Q3*^. (**C**-C””) InR^INTRA^-CFP (C’) and Chico-PH*-RFP (C”) in wild-type tissue. (**D**-D””) InR^INTRA^-CFP (D’) and Chico-PH*-RFP (D”) in *lnk*^*−/−*^. (**E**-E”’) Model of Lnk function in IIS. In wild-type tissue, Lnk ensures enrichment of InR and Chico at the cortical membrane (**E**). In the absence of Chico, the p60 subunit of PI3K is able to directly bind to InR (E’). In *lnk* mutants, despite the reduction of InR at the cellular membrane, Chico is able to bind to residual InR; hence the IIS pathway is partially active (E”). By contrast, when Lnk and Chico are lacking, p60 localisation by the residual InR at the membrane is not sufficient to promote pathway activity (E”’). Number of samples analysed: (**A**) n=8, (**B**) n=7, (**C**) n=7, (**D**) n = 6. Scale bars represent 50 μm.

Finally, we investigated the interaction between InR-CFP and Chico-RFP in a *lnk* mutant background. Similarly to Lnk, Chico-RFP was able to recruit InR^INTRA^-CFP to the membrane, either in a wild-type (Figure 4A and Additional file 1: Figure S1C) or in a *lnk* mutant (Figure 4B and Additional file 1: Figure S1C) background, although a significant proportion of InR^INTRA^-CFP and Chico-RFP remained in the cytoplasm when Lnk was lacking. However, when InR^INTRA^-CFP and Chico-PH*-RFP were overexpressed in *lnk* mutant salivary glands, the membrane enrichment of both was abolished (Figure 4D and Additional file 1: Figure S1C) in contrast to the situation where Lnk was present (Figure 4C and Additional file 1: Figure S1C). Thus, Lnk is required to reinforce the InR-Chico interaction at the membrane.

## Conclusions

By combining genetics with in vivo localisation studies on InR and its two adaptor molecules, Chico and Lnk, we gained insight into the molecular mechanisms at the plasma membrane that ensure proper IIS activation. Our data support the following model: Lnk is required to enrich InR and Chico at the plasma membrane (Figure 4). In a *lnk* mutant situation, fractions of InR and Chico are still localised at the cortical membrane, due to their transmembrane and PH domains, respectively. Thus, the IIS pathway is partially active in the absence of Lnk (Figure 4E”). In *chico* mutants, InR is capable of directly interacting with PI3K [28]; hence *chico* mutants are viable (Figure 4E’). By contrast*, chico; lnk* double mutants are lethal, probably due to mislocalisation and/or instability of InR, resulting in an insufficient signal from the receptor to PI3K (Figure 4E”’). Mutations in *lnk* weaken the InR-Chico interaction, reducing the capability of InR to phosphorylate Chico, as it was observed by Song and colleagues [16]. Future studies should aim at elucidating the precise mechanism of how Lnk promotes the InR-Chico interaction. It remains to be determined whether Lnk is required for trafficking, localisation at specific subdomains of the cortical membrane or stabilisation of InR and/or Chico.

## Methods

### Lnk, chico and InR constructs

PCRs from *Drosophila lnk*, *chico* and *InR* genes were performed using primers listed in Additional file 2: Table S1. Point mutations introduced in the PH domains of Lnk and Chico were C254Y and W100L, respectively [17,29]. PCR products were cloned into pENTR-TOPO (Invitrogen). Subsequent Gateway reactions were performed to shuffle the sequences into modified pUASTattbCFP and pUASTattbRFP vectors.

The pUASTattb vector was modified in order to introduce CFP or RFP coding sequences. pUASTattb and pAWC or pAWR (obtained from Drosophila Genomics Resource Center) were digested with NheI and NotI to swap the Gateway cassette from the pUASattb plasmid to pAWC or pAWR cassettes, respectively, to introduce CFP or mRFP coding sequences downstream of the attR2 sites.

### Fly transgenes and mutants

Mutant alleles used were *lnk*^*4Q3*^[17] and *chico*^*1*^. *hsp-Gal4* and *GMR-Gal4*[30] were used to drive expression of the transgenes. Vectors carrying *UAS-InR*, *InR*^*INTRA*^, *chico* or *lnk* were injected in *ZH-attP-86Fb* or *ZH-attP-44F* (*InR*^*INTRA*^) embryos [31]. The functionality of the tagged proteins was confirmed as follows (Additional file 3: Figure S2): Overexpression of *UAS-InR-CFP* using the *GMR-Gal4* driver [30] resulted in a significant overgrowth of the eyes as compared to *GMR-Gal4 UAS-GFP* control flies. Overexpression of *UAS-chico-RFP* and *UAS-lnk-CFP* (by means of MARCM clones) in eye imaginal discs promoted higher phospho-PKB levels (as a readout for IIS activity). The *tGPH* line was used as a reporter for IIS activity [18]. *UAS-Delta-RFP* (Bloomington 26696) [27] was used to perform the negative controls for the FRET experiments.

### Sample processing, FRET analyses and quantification of sublocalisation

Transgenic lines were crossed to *hsp-Gal4*. Third instar larvae were incubated at 37°C for 1 h. After 45 min of recovery, salivary glands were dissected in PBS and incubated for 15 min in Schneider’s medium with or without insulin (100 nM). After insulin treatment, salivary glands were fixed in 4% paraformaldehyde.

To examine interactions between Lnk, Chico and InR proteins, the FRET sensitised emission method was used. CFP was utilised as donor molecule and mRFP as acceptor molecule. FRET was analysed using a Leica SP2-AOBS confocal microscope. The FRET values were corrected for background fluorescence and crossover of donor and acceptor fluorescence. Corrected FRET was calculated as FRETc = FRET - (a x CFP) - (b x RFP)/RFP, with a and b representing the fractions of bleed-through of CFP and RFP fluorescence, respectively, through the FRET filter channel [32]. These values are presented as FRET efficiency (FRETeff). FRETeff values were averaged from regions of interest (ROIs) observed in cells from three independent experiments (n > 10) per condition and represented as mean ± standard deviation. For our sensors, we considered FRETeff ≥10% as positive FRET [33-35]. However, it is generally accepted that absence of FRET yields values <3%. To determine the percentage of samples (tissues) with positive FRET, we considered any ROI with FRETeff ≥10% after scanning regions where the two constructs co-localised as positive. After applying Gaussian Blur filter (Sigma: 2) with ImageJ software, FRET^c^ was presented in pseudocolour mode according to a temperature-based Look Up Table (LUT) with blue (cold) indicating low values and red (hot) indicating high values. LUT was linear, covering the full range of the data.

To quantify the subcellular distribution of the tagged proteins, the Plot Profile function from ImageJ software was used. Pixel intensities at the membrane and in the cytoplasm were measured across the cell (at least two cells were measured in each tissue sample), avoiding the nuclei and taking Actin pixel intensity as indicator for the membrane sub-compartment. Pixel intensities were averaged for each fraction, and the ratio between the means was calculated as a measure for the relative amount of protein per sub-compartment. The R package was used to perform one-tail *t*-test statistical analyses and boxplots.

### Immunostainings and clonal analysis

For dominantly marked clones (MARCM system), *FRT40 chico*^*1*^ or *FRT40iso* and *y w hs-flp UAS-GFP; tub-Gal80 FRT40/CyO y*^*+*^*; tub-Gal4/TM6B* flies were used. Clones were induced in second instar larvae (48 h after egg deposition (AED)) at 37°C for 15 min (dissection 96 h AED).

Rabbit anti *Drosophila* phospho-Akt/PKB Ser505 (1:300, Cell Signaling) staining was carried out on eye imaginal discs. Discs were dissected in PBS and fixed in 4% paraformaldehyde for 20 min at room temperature. After blocking (PBS, 0.3% Triton X-100, 2% NDS), imaginal discs were incubated with primary antibody at 4°C overnight. Goat anti-rabbit-Cy3 (1:200, Molecular Probes) was used as secondary antibody for 2 h at room temperature. AlexaFluor 647 phalloidin (1:40, Molecular Probes) was used for Actin staining. Nuclei were stained with DAPI before mounting in Vectashield. Samples were captured using a Leica SPE TCS confocal laser scanning microscope. Images were processed using NIH ImageJ software. Final artwork was prepared using Adobe Photoshop CS5 and Illustrator.

## Competing interests

The authors declare that they have no competing interests.

## Authors’ contributions

IA, EH and HS conceived the experiments. IA and IP performed the experiments. IA analysed the data. IA and HS wrote the paper. All the authors read and approved the final manuscript.

## Supplementary Material

Additional file 1: Figure S1 Summary of subcellular localisation analyses. (**A**) Boxplots and data summary related to Figure 2. (**B**) Boxplots and data summary related to Figure 3. (**C**) Boxplots and data summary corresponding to Figure 4. Click here for file

Additional file 2: Table S1 Primer sequences used to generate pENTR vectors for subsequent Gateway cloning. Click here for file

Additional file 3: Figure S2 InR-CFP, Chico-RFP and Lnk-CFP fusion proteins promote IIS activity. (**A-C**) Overexpression of *UAS-InR-CFP* using the *GMR-Gal4* driver (**B**) results in overgrown eyes as compared to the control (*UAS-GFP*, **A**). (**C**) Eye areas of *GMR > InR-CFP* and *GMR > GFP* flies (n = 13, p < 0.01). (**D**-D””) MARCM clones in eye discs overexpressing *UAS-Chico-RFP* show an increase in phospho-PKB levels (D” and D””). (**E**-E””) MARCM clones overexpressing *Lnk-CFP* (E’) exhibit increased phospho-PKB levels (E” and E””). Scale bars represent 100 μm (**A** and **B**) and 50 μm (**D** and **E**), respectively. Click here for file
