# Supplementary figures and images for: The Lnk/SH2B adaptor provides a fail-safe mechanism to establish the Insulin receptor-Chico interaction
Source: Cell Commun Signal. 2013 Apr 16;11:26. doi: 10.1186/1478-811X-11-26 (PMC3637499; doi:10.1186/1478-811X-11-26)

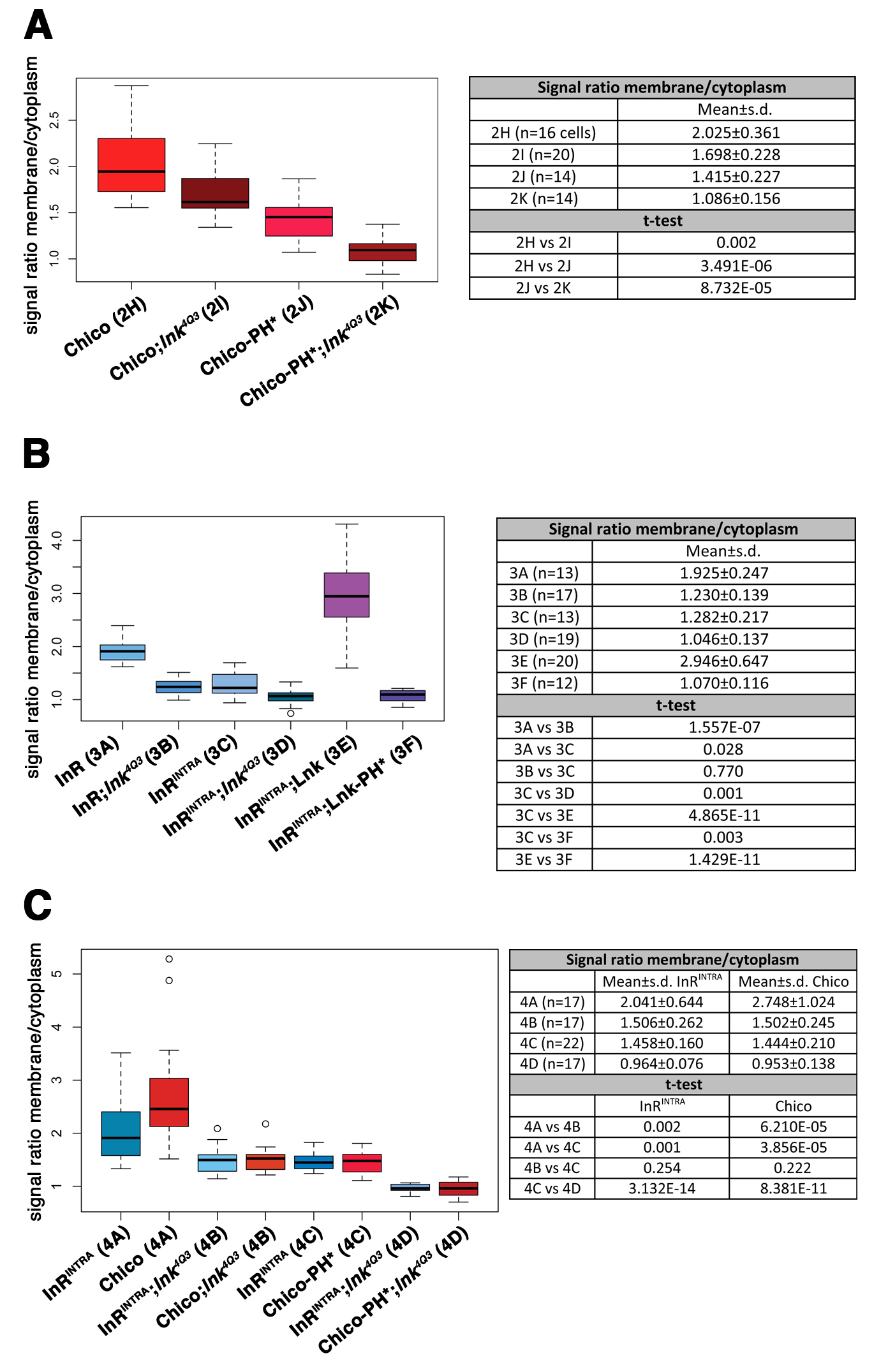

Supplement: Additional file 1: Figure S1 — Summary of subcellular localisation analyses. (A) Boxplots and data summary related to Figure 2. (B) Boxplots and data summary related to Figure 3. (C) Boxplots and data summary corresponding to Figure 4. [file 1478-811X-11-26-S1.jpeg]

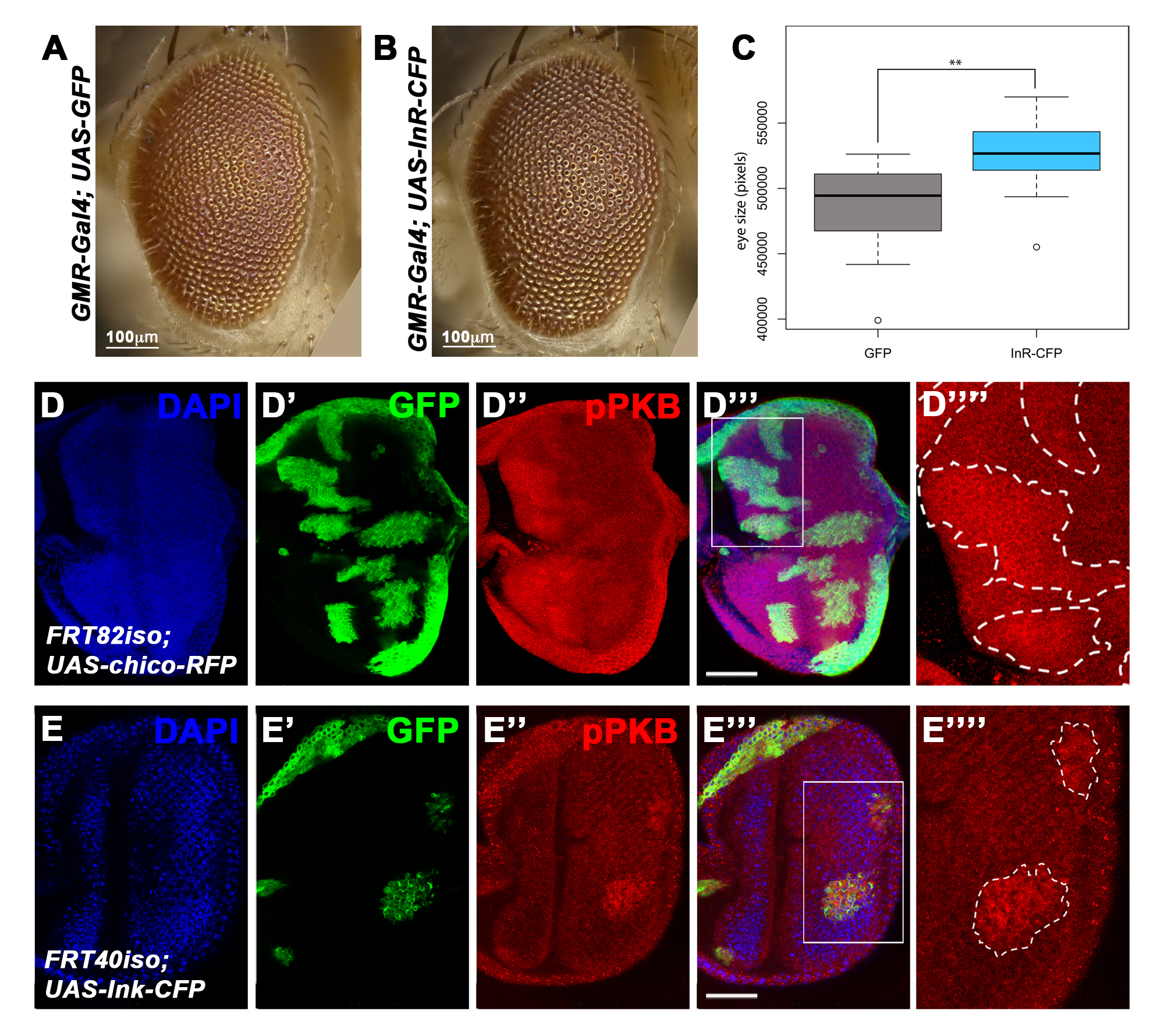

Supplement: Additional file 3: Figure S2 — InR-CFP, Chico-RFP and Lnk-CFP fusion proteins promote IIS activity. (A-C) Overexpression of UAS-InR-CFP using the GMR-Gal4 driver (B) results in overgrown eyes as compared to the control (UAS-GFP, A). (C) Eye areas of GMR > InR-CFP and GMR > GFP flies (n = 13, p < 0.01). (D-D””) MARCM clones in eye discs overexpressing UAS-Chico-RFP show an increase in phospho-PKB levels (D” and D””). (E-E””) MARCM clones overexpressing Lnk-CFP (E’) exhibit increased phospho-PKB levels (E” and E””). Scale bars represent 100 μm (A and B) and 50 μm (D and E), respectively. [file 1478-811X-11-26-S3.jpeg]
